# DNA polymerase characteristics influence noise levels in sequencing of short tandem repeats

**DOI:** 10.1186/s12864-026-12985-4

**Published:** 2026-05-26

**Authors:** Tova Lindh, Maja Sidstedt, Kevin M. Kiesler, Peter M. Vallone, Johannes Hedman

**Affiliations:** 1 Division of Biotechnology and Applied Microbiology, Department of Process and Life Science Engineering, Lund University, Lund, SE-221 00 Sweden; 2 National Forensic Centre, Swedish Police Authority, Linköping, SE-581 94 Sweden; 3 Applied Genetics Group, National Institute of Standards and Technology, 100 Bureau Drive, M/S 8314, Gaithersburg, MD 20899 USA

**Keywords:** Base substitutions, Fidelity, in vitro polymerization, PCR, Processivity, STR, Stutter, Sequencing

## Abstract

**Background:**

Polymerase chain reaction (PCR) applications including sequencing rely on thermostable DNA polymerases and their ability to generate accurate amplicons. Polymerization errors may hinder the detection of low-level DNA variants such as mutations in clinical samples or DNA from minor contributors in crime scene traces with DNA from multiple individuals. Short Tandem Repeat (STR) markers are affected by both random base substitutions and stutter, i.e., products which have lost or gained repeat units. The mechanisms leading to stutter formation have not yet been fully elucidated.

**Results:**

Here, we applied an STR assay based on Unique Molecular Identifiers to study the effects of DNA polymerases with different characteristics on amplicon yield and formation of PCR errors. The levels of base substitutions were clearly connected to the fidelity of the DNA polymerases, which in turn was coupled with having an integrated 3’ to 5’ exonuclease domain. Stutter formation was not associated with fidelity. DNA-binding domains improve processivity, which in turn has been suggested to lower the incidence of stutter. However, no such effect was seen in the present study as a polymerase having a DNA-binding domain gave the highest stutter levels.

**Conclusions:**

Overall, the degree of stuttering is likely due to several different DNA polymerase characteristics affecting the stability of the ternary complex and extension kinetics. This study highlights the importance of an increased understanding of DNA polymerase function and how this can influence the quality of the sequencing results, especially when analyzing complex parts of the human genome such as STR markers.

**Graphical abstract:**

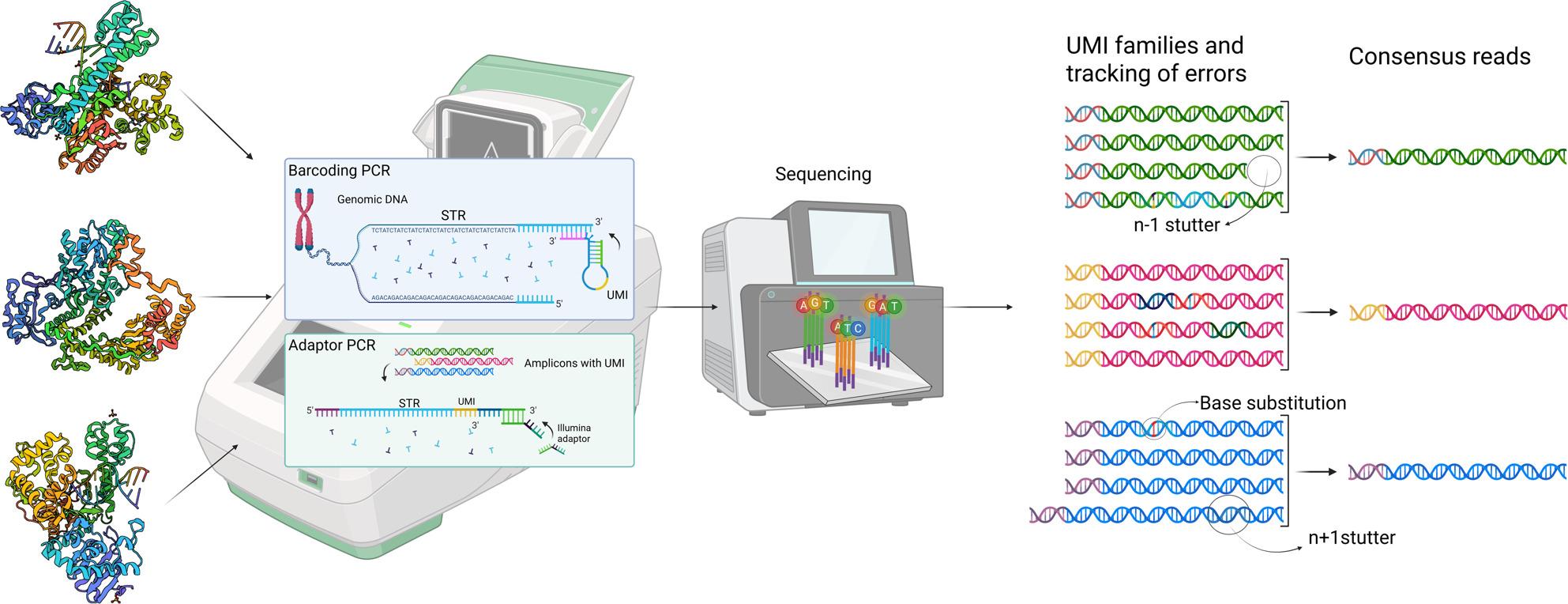

**Supplementary Information:**

The online version contains supplementary material available at 10.1186/s12864-026-12985-4.

## Introduction

Polymerase Chain Reaction (PCR) has revolutionized molecular biology by enabling sensitive detection and analysis of specific DNA sequences. These features are fundamental for applications ranging from genetic research to diagnostics and forensic science. However, despite its widespread use, PCR is not without limitations. The analysis of low quantities or partly degraded DNA is challenging [1, 2], and the applied thermostable DNA polymerases struggle in the presence of inhibitory compounds from the samples or sample matrices [3, 4]. Additionally, thermostable DNA polymerases form amplicons containing polymerization errors [5]. PCR inhibitors and low DNA quantity and quality generally lead to loss of information, whereas erroneous amplicons may obscure the true sequence and either hinder detection or result in false signals [3, 6].

The choice of thermostable DNA polymerase greatly affects the ability to obtain consistent and correct results in sequencing applications [7]. DNA polymerase characteristics impact both the accuracy of the analysis and the yield of amplicons [8]. Most library preparation methods utilize high-fidelity proofreading DNA polymerases with 3’-5’ exonuclease activity with the hope of obtaining reliable sequence data with a minimum of base substitution errors. Commercial and engineered DNA polymerases of that type, such as Phusion High Fidelity DNA polymerase and KAPA HiFi, have been found to result in relatively low levels of single-base substitutions [8, 9].

Microsatellites or Short Tandem Repeats (STR) are polymorphisms that consist of repeated motifs, generally 1 to 6 base pairs in size, and make up around 3% of the human genome [10, 11]. STRs have formed through evolution via recurring insertions and deletions of particular motifs [12]. Over human generations, slowly accumulated mutations have led to differing numbers of repeats between individuals. This has made STR markers suitable for parental and relationship DNA testing as well as for identification of individuals in criminal investigations, where they have been widely employed for three decades [13]. Some STR variants are connected with diseases such as neurological disorders or cancer and may thus be targeted in clinical diagnostics [14, 15]. The mutation rate for insertions and deletions of repeats (“stutter”) is typically higher than the rate of point mutations [12]. The mechanism behind the formation of stutter variants in vivo is mainly slipped-strand mispairing during replication due to the repetitive nature of STRs [16]. Smaller repeat motifs generally have higher mutation rates than larger ones, e.g., dinucleotides stutter more than trinucleotides [17].

 In vitro, i.e., when performing STR analysis using PCR, the propensity to form stutter amplicons differs between STR markers depending on the sequence of the repeat motif and the number of uninterrupted identical repeats [18]. Thus, a larger number of repeats leads to an elevated stutter risk. For a specific marker, most stutters thus affect the longest uninterrupted stretch (LUS) of repeats [18]. However, stuttering in other segments than LUS also occurs at detectable levels [19]. Additionally, polymorphisms in the flanking regions nearest the STR, i.e., indels or SNPs, have been shown to affect stutter formation [20], e.g., through elevating the actual number of successive repeats.

Stutter artefacts are prevalent in STR analysis. The most abundant type of stutter is n-1, i.e., molecules one repeat shorter than the true allele. The n-1 stutter ratios typically range from 2 to 10% for commercial STR kits with capillary electrophoresis detection [21]. Stutters are systematic errors that follow a specific pattern, whereas base substitutions are caused by random events and may affect any base in the sequence of interest. Similar to the in vivo situation, base substitutions are substantially less common than stutters in vitro. These two different types of PCR errors may call for different solutions, e.g., in terms of DNA polymerase characteristics. Thus, it may be challenging to control and limit both at the same time.

The mechanisms leading to different proportions of stutters for different types of thermostable DNA polymerases have not been fully elucidated. Fidelity is a measure of how often a polymerase incorporates the wrong base and is generally presented as a frequency or in relation to Taq DNA polymerase (e.g., 20X implying that the given polymerase has a 20 times lower base substitution rate compared to Taq polymerase). The connection between fidelity and stutter formation is ambiguous. In one study, a Taq-based polymerase, lacking 3’-5’ exonuclease activity, was found to produce fewer stutter artefacts than two high-fidelity polymerases, and equal amounts as a third one [22]. High processivity may reduce stutter generation, as addition of a single-stranded DNA-binding protein from *Escherichia coli* (SSB) was shown to prevent polymerase slippage in vivo [23]. Additionally, a Taq polymerase fused with a bacteriophage T7 thioredoxin binding domain and the polypeptide thioredoxin gave a conformational change that led to substantially reduced stuttering in vitro [24]. Strand-displacement capability has also been shown to lower the level of stutters, as witnessed by the *Thermococcus litoralis* DNA polymerase which has inducible strand-displacement activity [23].

The high temperature for polymerization in PCR may affect stutter formation, as it may lead to a risk of denaturation of the primer-template complex and subsequent “slippage” and re-annealing in the stutter position [25]. Lowering the extension temperature has been shown to reduce stutter formation using either thermostable [26] or thermolabile DNA polymerases [25, 27], but neither method has found widespread use. PCR-free analysis is a potential way to reduce or remove stutters and single-base substitutions since polymerization errors propagate through the exponential growth of amplicons in PCR. Indeed, PCR-free library preparation has been shown to lower the incidence of stutter by ninefold compared to PCR protocols [18]. However, this approach calls for high amounts of DNA, limiting the applicability for single-cell analysis and other low-template samples.

Other than modifying the conditions in PCR or removing the PCR step, stutter artefacts and base substitutions may be handled informatically. One such strategy is to apply unique molecular identifiers (UMIs). There, UMIs containing a certain number of bases are used to label individual template molecules and generate consensus reads post analysis, theoretically removing polymerization errors. UMI labelling has been shown to reduce the impact of random base substitutions substantially, thus enabling the detection of rare variants in diagnostic and forensic samples [28]. Recently, UMIs have also been shown to lower the amount of stutter artefacts in STR analysis [29, 30]. For example, UMI labelling using the SiMSen-Seq STR analysis method resulted in a tenfold reduction of artefacts [30].

The formation of polymerization errors may depend on the type of template used by the DNA polymerase. In the initial PCR cycles, large genomic DNA molecules serve as the main template, whereas later cycles are dominated by re-amplification of STR amplicons of around 100 to 300 base pairs in size. Using traditional PCR, it is not possible to determine whether polymerization errors arise in the first few cycles or later in the process. Efforts have been made to study events in the first PCR cycles using chamber digital PCR [31], but this model is not readily transferable to STR analysis. Applying UMIs, e.g., with the SiMSen-Seq STR method [30], may enable the study of polymerization errors in the different parts of PCR separately. SiMSen-Seq STR library preparation is made up of two PCR steps: barcoding PCR and adaptor PCR. In the barcoding PCR, a low number of PCR cycles is used to label individual molecules with UMIs. The template is the input genomic DNA, i.e., large DNA molecules. In the adaptor PCR, only the complete amplicons from barcoding PCR serve as template. Each original amplicon is labelled with a unique UMI, making it possible to track errors occurring for amplification of this template molecule.

Here, we applied a previously developed SiMSen-Seq STR assay [30] to study the effects of DNA polymerases with different properties on the amplicon yield as well as the formation of PCR errors. Six DNA polymerases with distinct characteristics in terms of e.g., fidelity and processivity and with or without additional features such as exonuclease activity or DNA-binding domains were chosen to identify any links between each fundamental property and the risk for generating stutter artefacts and single-base substitutions. The different polymerases were evaluated in both PCR steps of the SiMSen-Seq library preparation, i.e., barcoding PCR and adaptor PCR. Thus, the impact on error formation of using genomic DNA (early cycles) or amplicons (later cycles) as template could be investigated by studying both the regular STR reads and the consensus reads. The distribution of reads within UMI families enabled the direct study of errors emanating from individual template molecules. This, in turn, provided a new depth in the understanding of how DNA polymerase characteristics are connected to the formation of stutters and single-base substitutions in STR analysis.

## Materials and methods

All work performed at NIST has been reviewed and approved by the NIST Research Protections Office (MML-16–0080). All work performed in Sweden has been approved by the Swedish Ethical Review Authority (2023-05921-1).

### Overview of the experimental design

**Fig. 1:**
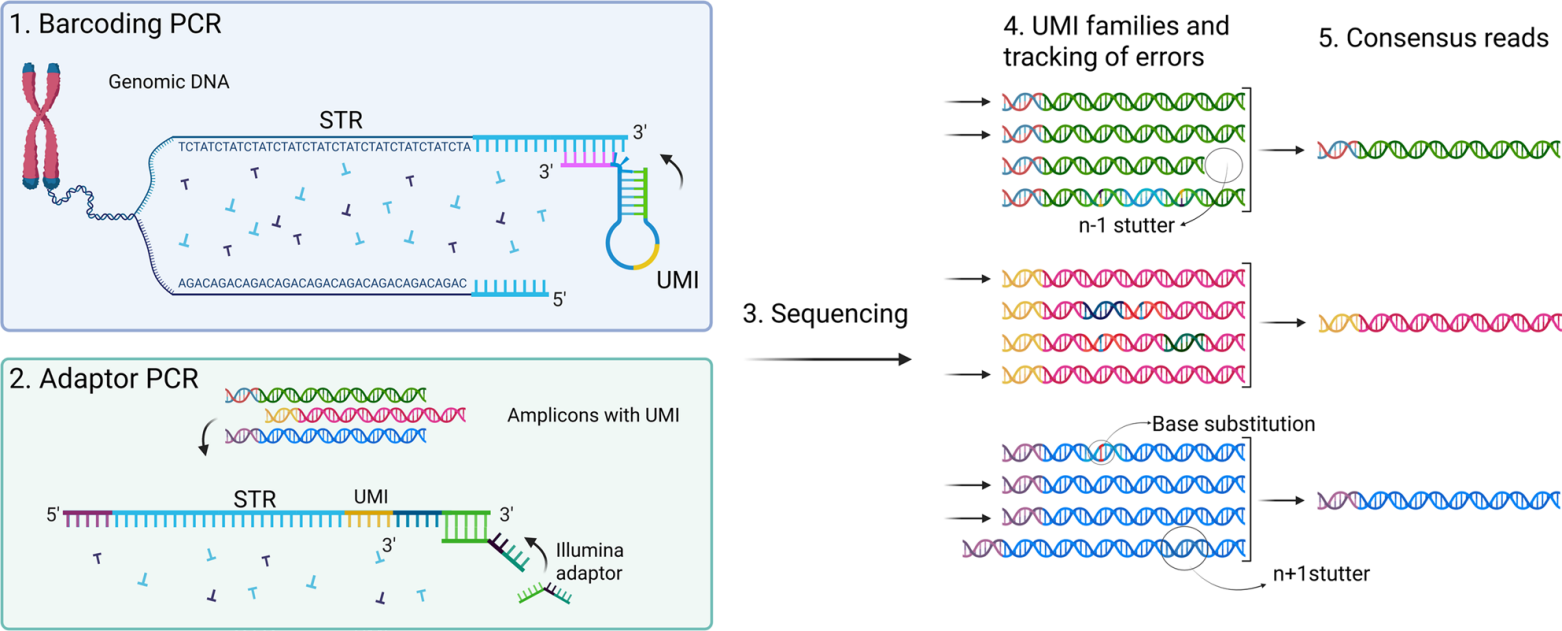
Schematic representation of the SiMSen-Seq STR workflow, illustrating UMI family construction and consensus read generation. The correct molecules are highlighted by arrows in step 4. Created with BioRender.com

The SiMSen-Seq 7-plex STR library preparation method with UMIs [28, 30] was used to compare the amplicon yield and levels of polymerization errors, i.e., stutter artefacts and single-base substitutions, for DNA polymerases with distinct characteristics. The initial barcoding PCR uses genomic DNA as input template (Fig. 1). There, three different polymerization processes give rise to targeted STR amplicons labelled with UMIs: (1) generation of long products, defined by the primer in one end, from genomic DNA, (2) generation of short products (STR amplicons) from long products and (3) generation of STR amplicons from other amplicons. The first two processes determine the overall efficiency of STR amplicon formation due to the low number of PCR cycles (four).

In the adaptor PCR, only STR amplicons from barcoding PCR serve as template. Thus, only the third process will take place, the generation of short products from other short products. Each short product STR amplicon is labelled with a certain UMI. All amplicons that stem from a specific short product comprise a UMI family, characterized by reads that share the same UMI (Fig. 1).

By using one DNA polymerase as a reference in barcoding PCR or adaptor PCR (i.e., SuperFi II), respectively, any differences between the tested DNA polymerases will be connected to events in either of the two PCR steps. Thus, it is possible to determine if the DNA polymerases are more or less prone to form stutters and single-base substitutions in the initial PCR cycles, using genomic DNA as template, or in the later cycles, using STR amplicons as template. Both STR reads and consensus reads generated from UMI families are studied.

Incorrect consensus reads may arise from an error introduced either during the barcoding PCR, or in an early cycle of the adaptor PCR. Studying the types of errors of incorrect consensus reads thus informs on the propensity of DNA polymerases to generate certain artefacts in the first few PCR cycles where genomic DNA is the dominant template. 50 UMI families with incorrect consensus sequences for each of the polymerases applied in the barcoding PCR were randomly selected for manual inspection. The UMI families were sorted into five different consensus read error categories, namely base substitutions, n-1 stutters, n-2 stutters, *n* + 1 stutters and *n* ± 0 stutters, where *n* ± 0 stutters refer to the simultaneous loss and gain of repeats in different repeat stretches.

A correct consensus read implies that the initial UMI labelled STR amplicon from barcoding PCR contained the correct sequence. In order to investigate errors originating from unique short product template molecules, we studied each sequence variant within UMI families with correct consensus reads and more than 40 members when applying different DNA polymerases in adaptor PCR. Five features previously used in a machine learning filter [30] were calculated to describe and compare UMI families between polymerases (see Bioinformatic processing and data analysis).

### SiMSen-Seq 7-plex STR assay

The SiMSen-Seq seven STR multiplex assay used in this study was developed by Sidstedt et al. [30]. The assay includes the following STR markers, ordered from the smallest amplicon size to the largest: D2S441, D1S1656, D3S1358, vWA, D8S1179, D21S11 and D12S391 (Table 1). The markers were chosen to include those with simple repeat structures (i.e., having only one repeat motif), compound markers (with two different motifs) as well as complex markers (with more than one repeat motif and interruptions of one or more bases). The theoretical maximum read lengths (excluding 28 bp for the UMI and stem-loop structure) for analysis of 2800 M DNA are 100 bp for D2S441, 137 bp for D1S1656, 139 bp for D3S1358, 159 bp for vWA, 232 bp for D8S1179, 233 bp for D21S11 and 241 bp for D12S391. The barcoding PCR primers are up to 96 bp, with one primer containing the UMI, the adapter-specific sequence and the stem-loop structure, and the other primer containing the adapter-specific sequence [32].

**Table 1:** Repeat structures, motifs and allele sizes of the seven applied STR markers

STR marker	Repeat structure	Repeat motif(s)	2800 M STR genotypes
D1S1656	Complex – interruptions	Most common: [CTAT] Other: [AC], [AT] etc.	AC[5]AT[1]CTAT[12] / AC[6]CTAT[11]
D2S441	Simple/Compound	Most common: [TCTA] Other: [TTTA]	TCTA[10] / TCTA[11]TTTA[1]TCTA[2]
D3S1358	Compound	[TCTA]1 [TCTG] 2–4 [TCTA]8–17	TCTA[1]TCTG[3]TCTA[14] / TCTA[1]TCTG[3]TCTA[13]
D8S1179	Compound	Most common: [TCTA] Other: [TCTG]	TCTA[1]TCTG[1]TCTA[12] / TCTA[2]TCTG[1]TCTA[12]
D12S391	Complex – interruptions	[TAGA], [CAGA]	TAGA[12]CAGA[6] / TAGA[15]CAGA[8]_+1T > C
D21S11	Complex – interruptions	Most common: [TCTA] Other: [TCTG]	TCTA[5]TCTG[6]TCTA[3]TATC[4]ATC[1]TATC[2]CATA[1]TCTA[11]TATC[2] / TCTA[4]TCTG[6]TCTA[3]TATC[4]A[1]TCTA[2]TCCA[1]TATC[12]
vWa	Complex	Most common: [GATA] Other: [GACA]	GATA[12]GACA[3]GATA[1] / GATA[14]GACA[4]GATA[1]

### Thermostable DNA polymerases

The following thermostable DNA polymerases from different sources and with different characteristics were applied in the study (Table 2): AccuPrime Pfx DNA polymerase (AccuPrime Pfx, Invitrogen, Waltham, MA, USA), AccuPrime Taq DNA polymerase High Fidelity (AccuPrime Taq HF, Invitrogen), TaKaRa ExTaq DNA polymerase Hot-Start version (ExTaq HS, TaKaRa Bio Inc., Kusatsu, Japan), Immolase (Bioline, Memphis, TN, USA), Phusion Hot Start II DNA polymerase (Phusion HS II, Thermo Scientific, Waltham, MA, USA) and Platinum SuperFi II DNA polymerase (SuperFi II, Invitrogen). In all comparisons between different DNA polymerases in barcoding PCR, SuperFi II was applied in the adaptor PCR, and vice versa. SuperFi II was chosen as a reference since it had previously been applied in SiMSen-Seq STR analysis [30].

**Table 2:** Specifications of the applied thermostable DNA polymerases

DNA polymerase	3’ – 5’ exonuclease activity	5’ – 3’ exonuclease activity	Hot start activation	Fidelity (vs. Taq polymerase)	Family	Source and type
AccuPrime Pfx	Yes	No	Platinum anti-Pfx DNA polymerase antibodies	26X	B	*Thermococcus kodakarensis* (KOD) polymerase
AccuPrime Taq HF	Yes	Yes	Platinum antibody complexes	9X	A	Blend of *Thermus aquaticus* polymerase and a proofreading enzyme
ExTaq HS	Yes	Yes	Antibody-mediated hot-start	4.5X	A	Blend of *Thermus aquaticus* polymerase and an exonuclease
Immolase	No	Yes	Molecule covalently bound to active site	1X*	A	Novel variant, undisclosed organism
Phusion HS II	Yes	No	Specific Affibody ligand	52X	B	*Pyrococcus furiosus* polymerase, with Sso7d DNA-binding domain
SuperFi II	Yes	No	Proprietary antibodies	> 300X	B	Engineered *Pyrococcus-*like polymerase with Sso7d-like DNA-binding domain

*Fidelity not specified by the manufacturer but likely similar to Taq DNA polymerase since it is a Family A polymerase without 3’-5’ exonuclease activity

The six DNA polymerases that were evaluated in this study were chosen to represent enzymes coming from different organisms and possessing distinct characteristics in terms of e.g., fidelity and processivity. In order to focus the evaluation as much as possible on the polymerase properties and not assay differences, we applied the same annealing temperature, Mg^2+^ concentration and thermal cycling times for all of them, while maintaining the enzyme-specific buffers.

### DNA samples

Reference DNA and single-source DNA samples were used in this study. The DNA materials used were 2800 M Control DNA (2800 M) (Cat. nr. DD7101, 10 ng/µL, Promega, Madison, WI, USA), a single-source male genomic DNA commonly applied as a control in STR analysis, NIST Standard Reference Material (SRM) 2391d Component C [33], a single-source male genomic DNA (1.6 ± 0.2 ng/µL), and ten well-characterized single-source samples from NIST with published or known STR profiles [34].

### Library preparation and sequencing

Barcoding PCR was performed with the following DNA polymerases: AccuPrime Pfx, AccuPrime Taq HF, ExTaq HS, Immolase, Phusion HS II and SuperFi II. Different batches of all reagents were used in the two laboratories. The following reagents and concentrations were used in a total reaction volume of 10 µL: 1X polymerase specific PCR reaction buffer, 2.5 mmol/L MgCl_2_ (Roche, Basel, Schweiz), 0.5 mol/L L-carnitine inner salt (Sigma-Aldrich, Burlington, MA, USA), 0.2 mmol/L dNTPs (Roche), 40 nmol/L to 100 nmol/L of each barcoding primer (DNA Ultramer oligomers with standard desalting from Integrated DNA Technologies (IDT, Coralville, IA, USA)), 0.1 U DNA polymerase. One ng of template DNA was added to each reaction. Cycling was performed on a VeritiPro Thermal Cycler (Thermo Fisher Scientific, Waltham, MA, USA) (Laboratory A) or a ProFlex PCR System (Thermo Fisher Scientific) (Laboratory B) using the following settings: 98 ˚C for 3 min, 4 cycles of [98 ˚C for 10 s, 59 ˚C for 6 min and 72 ˚C for 30 s], 72 ˚C for 30 s and hold at 4 ˚C. Immolase required a 10 min heat activation step at 98 ˚C, instead of the 3 min used for the other polymerases. The ramp rate during cycling was set to 6.0 ˚C/s for the VeritiPro and 3.5 ˚C/s for the ProFlex.

The second step in library preparation was the adaptor PCR. The reaction mix was prepared accordingly, with a total reaction volume of 25 µL: 1X polymerase specific PCR reaction buffer, 2.5 mmol/L MgCl_2_ (Roche), 0.5 mol/L L-carnitine inner salt (Sigma-Aldrich), 0.2 mmol/L dNTPs (Roche), 0.4 µmol/L of each primer (HPLC purified oligomers, IDT), 1.0 U DNA polymerase. Additionally, 8 µL of the barcoding PCR reaction mixture was added as template. Cycling was performed on a VeritiPro Thermal Cycler (Thermo Fisher Scientific) (Laboratory A) or a ProFlex PCR System (Thermo Fisher Scientific) (Laboratory B) using the following settings: 98 ˚C for 2 min, 27 cycles of [98 ˚C for 10 s, 80 ˚C for 1 s, 72 ˚C for 30 s, 76 ˚C for 30 s] and hold at 4 ˚C. Immolase required a 10 min heat activation step at 98 ˚C, instead of the 2 min used for the other polymerases. The ramp rate during cycling was set to 0.4 ˚C/s.

After the adaptor PCR, purification of the products was performed with AMPure XP Beads (Beckman Coulter, Brea, CA, USA) at a 0.8X ratio vol/vol. Before adding the beads (28 µL), the reaction volume was adjusted to 35 µL by the addition of 10 µL nuclease-free water to the adaptor PCR reaction mixture. The protocol from the manufacturer was used, and the final products were eluted in 20 µL Tris-EDTA buffer (0.1 mM EDTA, pH 8.0; Quality Biological, Gaithersburg, MD, USA). The purified libraries were analyzed with either Fragment Analyzer System 5200 (Agilent Technologies, Santa Clara, CA, USA) using the HS Small Fragment kit (50–1500 bp, Product nr. DNF-477-0500) (Laboratory A) or the TapeStation 4150 (Agilent) and High Sensitivity D1000 Reagents (Product nr. 5067–5584–5587, Agilent) (Laboratory B) as quality control before sequencing.

Thereafter, DNA concentrations were determined using the Qubit 1X dsDNA HS Assay Kit (Product nr. Q33230, Thermo Fisher Scientific). Libraries were equimolarly normalized and diluted to 4 nmol/L in one pool. The pool was diluted to 8 pmol/L with ~ 10% PhiX (Illumina, San Diego, CA, USA) spike-in. Sequencing was performed on MiSeq FGx instrument (Qiagen, Hilden, Germany) at two separate laboratories (Laboratory A and B) using the MiSeq Reagent Kit v3, 600 cycles (Illumina) in 2 × 300 cycles paired-end read mode.

### Bioinformatic processing and data analysis

The data was processed using the bioinformatic pipeline UMIec Forensics (available at https://github.com/agynna/UMIec_forensics), previously developed by Sidstedt et al. [30]. In short, paired-end reads were trimmed with AdapterRemoval [35] and merged with a modified version of FLASH [36]. Reads were assigned to STR markers using FDSTools TSSV [37] and grouped into UMI families by UMIErrorCorrect [38], whereafter one consensus read per UMI family was generated (Fig. 1). Consensus reads were generated by taking the most common sequence for each UMI. Only UMI families with three or more members were considered for generation of consensus reads. STR alleles were called from the consensus reads using FDSTools v. 2.0.4 [37]. Each sample was also analyzed in parallel with FDSTools omitting the UMI information.

The data was summarized at different levels to study effects both with the UMIs and without using the UMI information. Here, raw reads refer to all the reads in the FASTQ file. The terms “read” or “STR read” are used for reads assigned to STRs and the term “consensus read” is used for sequences that have been generated from UMI families.

After UMI family construction, five features were calculated to further evaluate the different sequences within the UMI families [30]. “Purity” was defined as the proportion of members identical to the most common sequence in the UMI family, i.e., the consensus read. “Proportion of n variants” was defined as the number of different sequence variants in the UMI family, divided by the total number of members. “Proportion minus one” was defined as the proportion of members one repeat unit shorter than the consensus sequence (n-1 stutter), “Proportion minus two” as the proportion of members two repeat units shorter than the consensus sequence (n-2 stutter) and “Proportion plus one” as the proportion of members one repeat unit longer than the consensus sequence (*n* + 1 stutter).

Data analysis and plotting was done in Python using the packages *matplotlib* (v. 3.8.3), *pandas* (v. 2.2.1), *scipy* (v. 1.12.0), *seaborn* (v. 0.13.2) and *statsmodels* (v. 0.14.4).

### Statistical analysis

The non-parametric Mann-Whitney U test was used for pairwise comparison to assess differences in raw read counts and proportion of recognized reads (assigned to STRs) between the laboratories for each of the DNA polymerases. This test was used due to its suitability for comparing two independent groups without assuming a normal distribution of the data. Where multiple comparisons were performed, p-values were adjusted using Benjamini-Hochberg procedure to control the false discovery rate (FDR). Statistical significance was defined as FDR-adjusted p-values less than 0.05. The non-parametric Kruskal-Wallis test followed by Dunn’s post hoc test was used to assess differences in error rates between the different DNA polymerases before and after UMI family generation and between the laboratories. This test was used due to its suitability for comparing multiple groups of data with unequal sample size, which do not meet the assumption of normally distributed data. All statistical analyses were performed in Python using the *scipy* and *statsmodels* packages.

## Results

Five sequencing runs were performed in two different laboratories utilizing different reagent batches, with 48 reactions per run. The quantity and quality of all libraries were verified before sequencing using fluorometry and fragment analysis (Supplementary Table S1). All sequencing quality metrics were acceptable, although cluster passing filter was a bit lower and pre-phasing and phasing were somewhat higher than the recommended values in some cases (Table 3). Such deviations are common when running high numbers of sequencing cycles and generally do not affect sequencing quality.

**Table 3:** Overall sequencing quality metrics for each sequencing run. Values are recommended to be within the following intervals: cluster density between 400 and 1650 K/mm^2^, cluster passing filter above 80%, phasing below 0.25 and pre-phasing below 0.15

Sequencing run nr	Laboratory	Instrument	Cluster density (K/mm^2^)	Clusters passing filter (%)	Read 1 phasing	Read 1 pre-phasing	Read 2 phasing	Read 2 pre-phasing
1	Lab A	MiSeq FGx	1424	85.82	0.16	0.13	**0.34**	0.13
2	Lab A	MiSeq FGx	1341	90.64	0.15	0.12	0.25	0.12
3	Lab A	MiSeq FGx	1150	90.03	**0.27**	0.11	**0.39**	0.13
4	Lab B	MiSeq FGx	1376	**75.64**	**0.31**	**0.33**	**0.26**	0.08
5	Lab B	MiSeq FGx	**1652**	**76.48**	**0.28**	0.07	**0.38**	0.06

Values outside the recommended intervals are indicated in bold

### DNA polymerase characteristics affect the STR amplicon yield

#### STR amplicon yield for different DNA polymerases in barcoding PCR

**Fig. 2:**
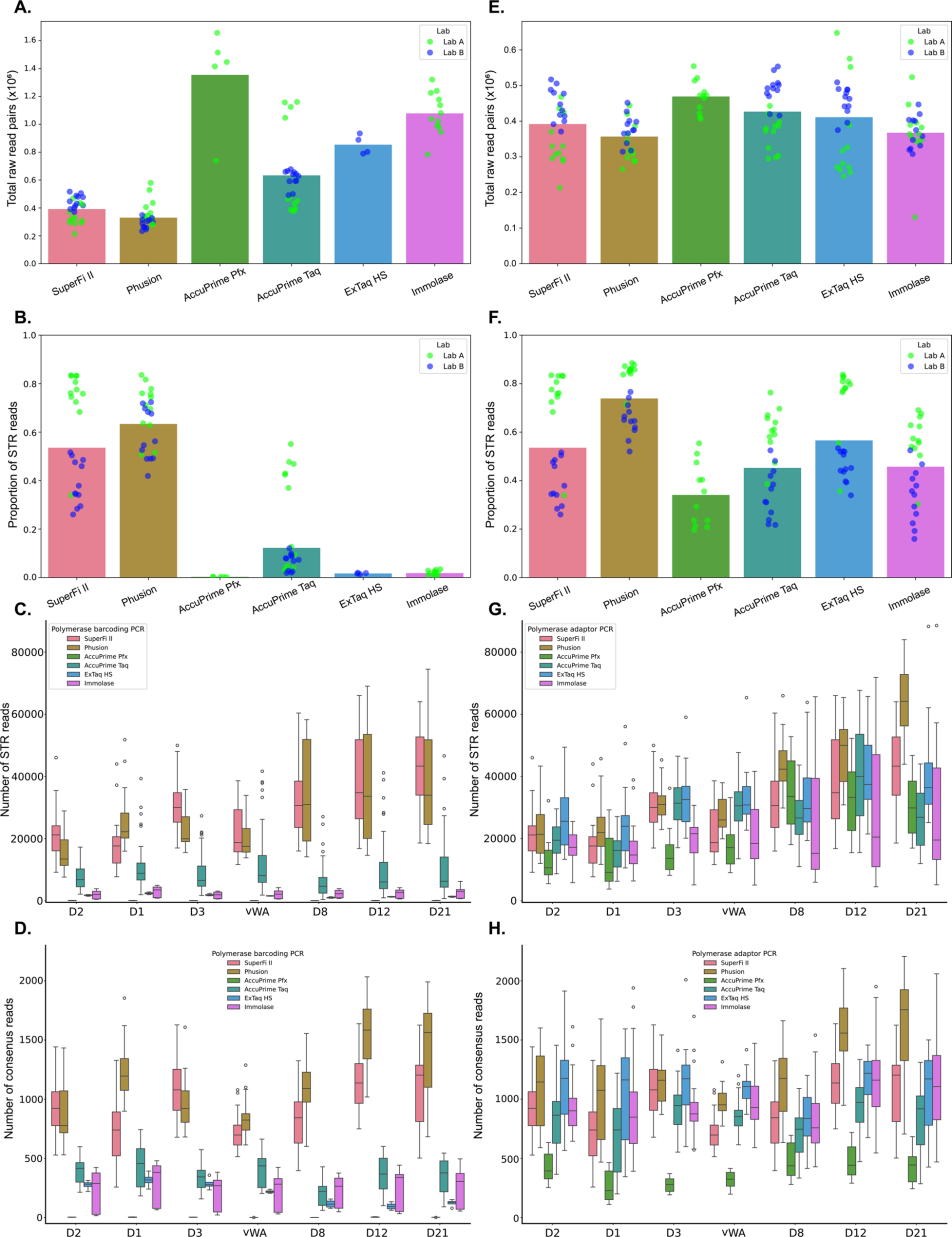
STR amplicon yield for different DNA polymerases in barcoding PCR (left column) and adaptor PCR (right column). **A** and **E** Number of paired raw reads for each of the polymerases applied either in the barcoding PCR (A) or in the adaptor PCR (E), where the color of the individual samples indicates the lab. **B** and **F** Proportion of reads assigned to STR markers (STR amplicon yield) for each polymerase in the barcoding PCR (B) and in the adaptor PCR (F). **C** and **G** Reads per marker for each polymerase in the barcoding PCR (C) and in the adaptor PCR (G). **D** and **H** Consensus reads per marker for each polymerase in the barcoding PCR (D) and in the adaptor PCR (H). SuperFi II was used in the barcoding PCR when studying the effects of different DNA polymerases on adaptor PCR, and vice versa. In A, B, C and D *n* = 24 for SuperFi II, Phusion HS II and AccuPrime Taq HF, *n* = 5 for AccuPrime Pfx, *n* = 4 for ExTaq HS and *n* = 12 for Immolase. In E, F, G, and H *n* = 24 for SuperFi II, Phusion HS II, AccuPrime Taq HF, ExTaq HS and Immolase and *n* = 12 for AccuPrime Pfx. In C, D, G and H.: the boxplots show median values, the first and third quartiles and the whiskers 1.5 interquartile ranges, dots represent outliers

Overall, there was a substantial variation in STR amplicon yield between the DNA polymerases applied in barcoding PCR (Fig. 2A and B). SuperFi II and Phusion HS II resulted in the lowest total numbers of raw reads (ranging from 210,000 to 580,000), but showed the highest selectivity, as 26 to 84% of the reads were assigned to STR markers. AccuPrime Taq HF generated an average of 630,000 raw reads per sample, with a range from 380,000 to 1,160,000. Of these, 1.9% to 55% were attributed to STR markers. For ExTaq HS and Immolase, 1.4 to 2.0% and 0.56 to 3.4% of the raw reads were STRs, respectively. AccuPrime Pfx yielded high numbers of raw reads, but almost no STR reads (between 0 and 0.34%). Fragment analysis showed that the unwanted amplicons were shorter than the STRs, suggesting that they mostly consisted of primer dimers and adapter dimers (Supplementary Figure S1). It was apparent that the barcoding PCR conditions, targeting genomic DNA with low amounts of polymerase and primers, and applying four PCR cycles, were challenging for some enzymes.

SuperFi II and Phusion HS II resulted in higher numbers of STR reads per marker than the other polymerases, ranging from 7,600 to 74,000 (Fig. 2C). AccuPrime Taq HF gave between 400 and 47,000 STR reads per marker, and Immolase resulted in less variation across samples and markers and yielded between 470 and 6,300. ExTaq HS, only used in four reactions, gave approximately 1000 reads per marker. AccuPrime Pfx consistently produced zero or near-zero reads per marker (between 16 and 145 reads).

When using the UMI information to generate consensus reads, SuperFi II and Phusion HS II gave between 260 and 2,000 consensus reads per marker (Fig. 2D). Immolase and AccuPrime Taq HF yielded fewer consensus reads, with mean values below 500 per marker. AccuPrime Pfx and ExTaq HS generated very low numbers of consensus reads (between zero and 400 per marker) and were excluded from evaluation of polymerization errors due to the low coverage.

#### STR amplicon yield for different DNA polymerases in adaptor PCR

In the adaptor PCR, the differences between the DNA polymerases were smaller in terms of both the total numbers of raw reads and the proportions assigned to STRs, compared to barcoding PCR (Fig. 2). Mean numbers of raw reads varied between 360,000 and 470,000, while individual samples ranged from 130,000 to 650,000 (Fig. 2E). The mean proportions of reads attributed to STR markers were between 34% and 75% across polymerases, with individual samples ranging from 16% to 88% (Fig. 2F). The low variation in both raw reads and proportions of STR reads between the different polymerases likely reflects the more favorable conditions in the adaptor PCR relative to the barcoding PCR. In adaptor PCR, higher amounts of DNA polymerase and higher primer concentrations were applied and STR amplicons from barcoding PCR served as template. All polymerases resulted in similar numbers of STR reads per marker (Fig. 2G). SuperFi II, Phusion HS II, AccuPrime Taq HF, ExTaq HS and Immolase all gave between 200 and 2,200 consensus reads per marker with moderate differences between the polymerases (Fig. 2H). AccuPrime Pfx gave substantially lower numbers of consensus reads. Generating STR amplicons with amplicons as template, and using reactions with excess of all reagents, reflects the conditions that manufacturers optimized their DNA polymerase-buffer systems for, and the similarities in performance are thus expected. Comparing the results obtained at the two laboratories revealed some differences in raw read counts and proportions of reads assigned to STR markers. When different polymerases were applied in the barcoding PCR, there were significant differences between the laboratories for SuperFi II and Phusion HS II (*p* ≤ 0.029), both in terms of raw read counts and proportions of STR reads (Fig. 2A and B). For AccuPrime Taq HF, only the proportion of reads assigned to STRs differed significantly (*p* = 0.037). In the adaptor PCR, significant differences between the two laboratories were seen for SuperFi II and AccuPrime Taq HF both in terms of raw read counts and proportions of STR reads (*p* ≤ 0.0029) (Fig. 2E and F). The proportion STR reads also differed significantly for Phusion HS II, ExTaq HS and Immolase (*p* ≤ 0.0008).

### Stutter artefacts and single-base substitutions for different DNA polymerases

#### PCR errors for different DNA polymerases in barcoding PCR

**Fig. 3:**
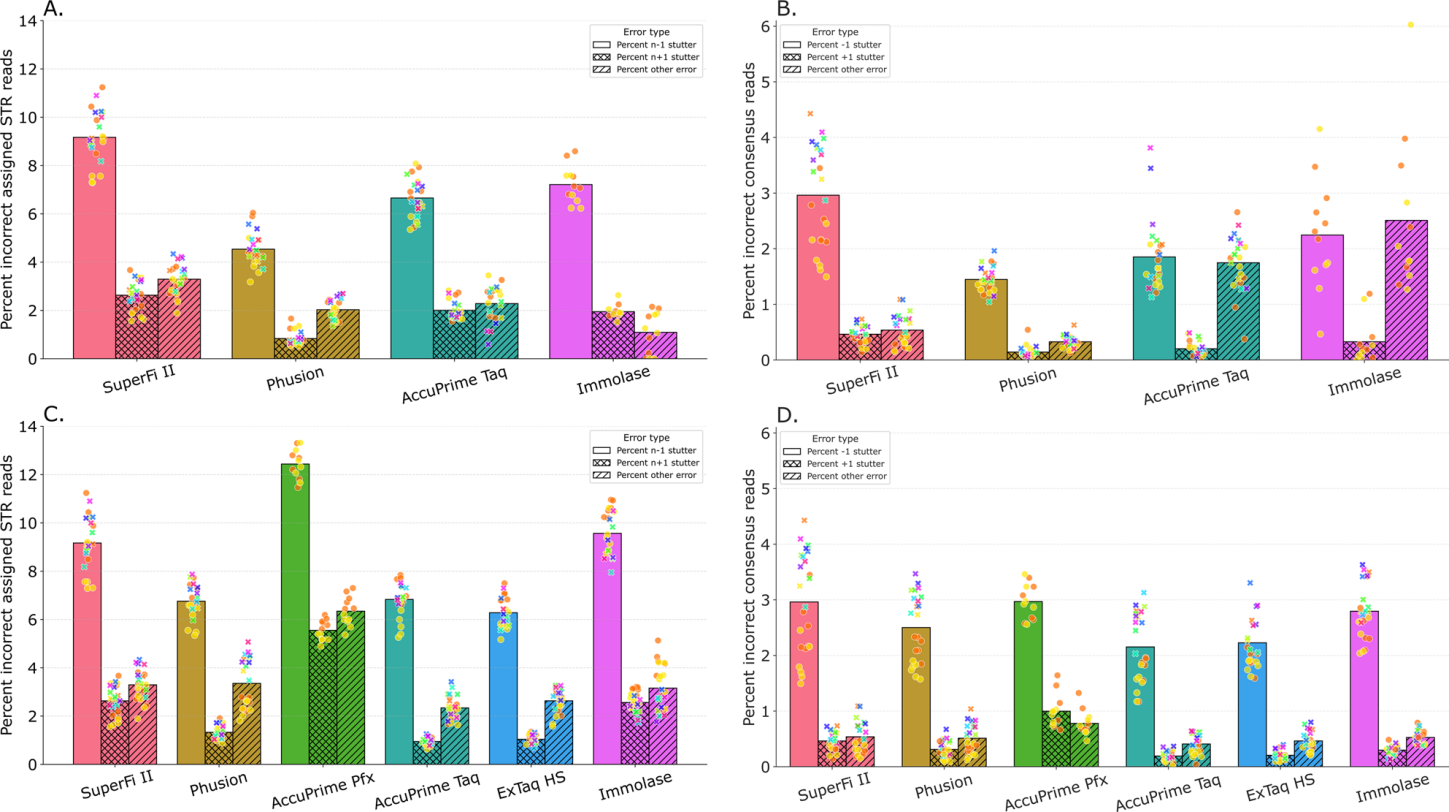
Proportions of errors in the barcode PCR (**A** and **B**) and adaptor PCR (**C** and **D**), for STR reads (left column), and consensus reads (right column). The proportions of errors are divided into n-1 stutters (left, no pattern), n+1 stutters (middle, crossed pattern), and other errors (base substitutions and rare stutter variants, right, striped pattern). The circles and crosses represent individual samples, where the circles were sequenced at Laboratory A and the crosses sequenced at Laboratory B. The different colors represent different DNA samples as input DNA. SuperFi II was used in the barcoding PCR when studying the effects of different DNA polymerases on adaptor PCR, and vice versa. In A and B n=24 for SuperFi II, Phusion HS II and AccuPrime Taq HF and n=12 for Immolase. In C and D n=24 for SuperFi II, Phusion HS II, AccuPrime Taq HF, ExTaq HS and Immolase and n=12 for AccuPrime Pfx

Four of the DNA polymerases applied in barcoding PCR (SuperFi II, Phusion HS II, AccuPrime Taq HF and Immolase) generated sufficient yields to study stutter artefacts and single-base substitutions for both STR reads and consensus reads. Regarding STR reads, Phusion HS II resulted in the lowest proportion of total errors, with 4.5 ± 0.7% of reads being n-1 stutters (Fig. 3A). Consensus read generation lowered the amount of n-1 stutters by more than three-fold, to 1.4 ± 1.1% (Fig. 3B). AccuPrime Taq HF followed with 6.7 ± 0.8% n-1 stutters for STR reads and 1.9 ± 1.6% for consensus reads, and Immolase gave 7.2 ± 0.8% for STR reads and 2.2% ± 2.1 for consensus reads. SuperFi II resulted in the highest error proportion among the polymerases, with 9.2 ± 1.1% n-1 stutters for STR reads and 3.0 ± 2.3% for consensus reads. 

**Fig. 4:**
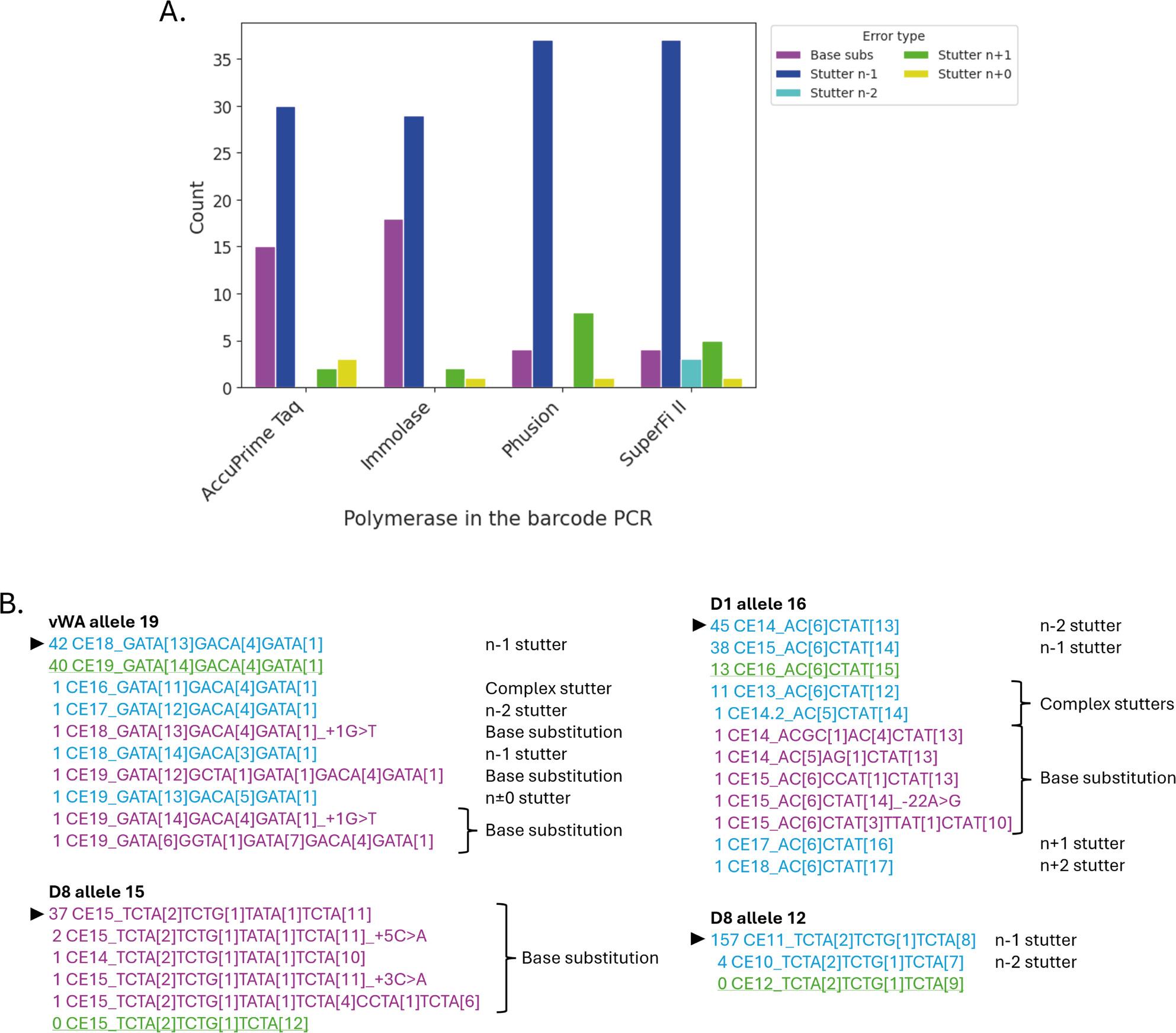
Results for 50 randomly selected UMI families with incorrect consensus reads for each polymerase used in barcoding PCR. **A** Distribution of error types among the incorrect consensus reads. **B** Examples of UMI families with incorrect consensus reads. The correct sequence is underlined (green) and the proposed cause of error indicated in blue (stutter artefacts) and purple (base substitutions). The most common sequence, i.e., the consensus read, is indicated by an arrow. Sequences are given in STRnaming format

Studying STR reads, n-1 stutters were roughly five times more common than *n* + 1 stutters and three times more common than other errors for all polymerases (Fig. 3A). Other errors ranged from a mean of 1.1% (Immolase) to 3.3% (SuperFi II). For consensus reads, the differences in overall proportions of errors were more pronounced across polymerases (Fig. 3B). There, AccuPrime Taq HF and Immolase resulted in 3.1 to 7.6 times higher proportions of other errors compared with SuperFi II and Phusion HS II (*p* ≤ 4.4∙10^− 6^). Through manual inspection of 50 UMI families with incorrect consensus reads for each of the DNA polymerases, it was confirmed that the higher levels of other errors for AccuPrime Taq HF and Immolase were mainly due to single-base substitutions (Fig. 4A). In some of the UMI families, the correct sequence was missing entirely. This implies that the error was introduced in barcoding PCR when a long PCR product was formed from the genomic DNA. Others contained the correct sequence, but it had fewer reads than the erroneous consensus sequence. Thus, the error occurred in barcoding PCR or very early in adapter PCR (see examples in Fig. 4B). AccuPrime Taq HF relies on a combination of a separate proofreading enzyme and Taq DNA polymerase and Immolase lacks proofreading activity, and both have lower fidelity compared to SuperFi II and Phusion HS II (Table 2). Significant differences between polymerases were also noted for n-1 and *n* + 1 stutters (Fig. 3B). For example, both Phusion HS II and AccuPrime Taq HF showed lower proportions of n-1 and *n* + 1 stutters than SuperFi II (p = ≤ 3.9∙10^− 4^), and Immolase gave less *n* + 1 stutter than SuperFi II (*p* = 4.6∙10^− 3^). These findings confirm that fidelity is directly linked to the incidence of base substitutions and that other properties may contribute to stuttering.

The stutter frequency was also impacted by the repeat structure of the specific marker (Table 1). D2 showed the lowest proportion of n-1 stutters across polymerases (below 2%) and D12 resulted in the highest (up to 12%) (Supplementary Figure S2). With Phusion HS II, a substantially lower proportion of n-1 stutter was observed for the marker D21 (mean 1%) compared to the other three enzymes (mean from 1.5% to 2%). Additionally, the results for D1 with SuperFi II showed higher n-1 stutter proportion than the other DNA polymerases (mean > 4% compared to about 2%).

When applying SuperFi II, there were some differences in levels of stutter artefacts and single-base substitutions between the laboratories for consensus reads. Sequencing performed at Laboratory B yielded significantly more errors and larger variation for all types of artefacts (*p* ≤ 2.4∙10^− 4^). In contrast, Phusion HS II and AccuPrime Taq HF (applied at both laboratories) produced consistent error levels across laboratories (*p* ≥ 0.37) (Fig. 3B). Different batches of all three DNA polymerases were used at the two laboratories.

#### PCR errors for different DNA polymerases in adaptor PCR

For all six DNA polymerases applied in the adaptor PCR, n-1 stutters were the most common error type, followed by other errors and *n* + 1 stutters. AccuPrime Pfx obtained the largest proportions of errors (mean n-1 stutters: 12.4 ± 0.6%: *n* + 1 stutters: 5.6 ± 0.4%; other errors: 6.3 ± 0.6%, p = ≤ 3.7∙10^− 2^ (except vs. Immolase *p* = 5.6∙10^− 2^)). SuperFi II and Immolase resulted in mean n-1 stutter percentages of 9.2 ± 1.1% and 9.6 ± 0.9%, respectively (no significant difference, *p* = 5.9∙10^− 1^), whereas Phusion HS II, AccuPrime Taq HF and ExTaq HS generated 6.8 ± 0.7%, 6.8 ± 0.7% and 6.3 ± 0.6% (no significant difference, p = ≥ 1.4∙10^− 1^) (Fig. 3C). The proportions of *n* + 1 stutters were similar for Phusion HS II (1.3%), AccuPrime Taq HF (0.95%) and ExTaq HS (1.0%), and substantially higher for SuperFi II (2.6%) and Immolase (2.6%) (p = ≤ 3.9∙10^− 2^). The mean percentages of other errors ranged from 2.3% to 3.6% for all polymerases except AccuPrime Pfx.

Following the UMI-based consensus read generation, the error proportions were substantially lowered across all polymerases (Fig. 3D). Also, the differences between the enzymes were reduced. However, especially for *n* + 1 stutters there were still significant differences between some polymerases. The more uniform results were expected since the consensus reads mainly represent the molecules generated by SuperFi II in barcoding PCR. The mean n-1 stutter proportions ranged from 2.2% (AccuPrime Taq HF) to 3.0% (SuperFi II and AccuPrime Pfx) and mean percentages of *n* + 1 stutters ranged from 0.19% (AccuPrime Taq HF) to 1.0% (AccuPrime Pfx). SuperFi II, Phusion HS II, ExTaq HS and Immolase showed similar amounts of *n* + 1 stutters (0.21–0.46%). For other errors, AccuPrime Pfx, excluded from the sequencing at Laboratory B, showed the largest proportion (mean 0.78%), while the other polymerases ranged from 0.41 to 0.54%. Examining erroneous consensus reads per marker showed a similar performance pattern to that observed when using different DNA polymerases in the barcoding PCR, with D2 exhibiting the lowest percentages of n-1 stutters and D12 the highest (Supplementary Figure S2).

There were significant differences (*p* ≤ 1.0∙10^− 3^) between the laboratories regarding proportions of consensus read errors for SuperFi II, Phusion HS II, AccuPrime Taq HF and ExTaq HS. No significant difference was seen for Immolase (*p* ≥ 0.15). Two different batches of all polymerases were used at the different laboratories.

### Studying PCR errors from individual template molecules

**Fig. 5:**
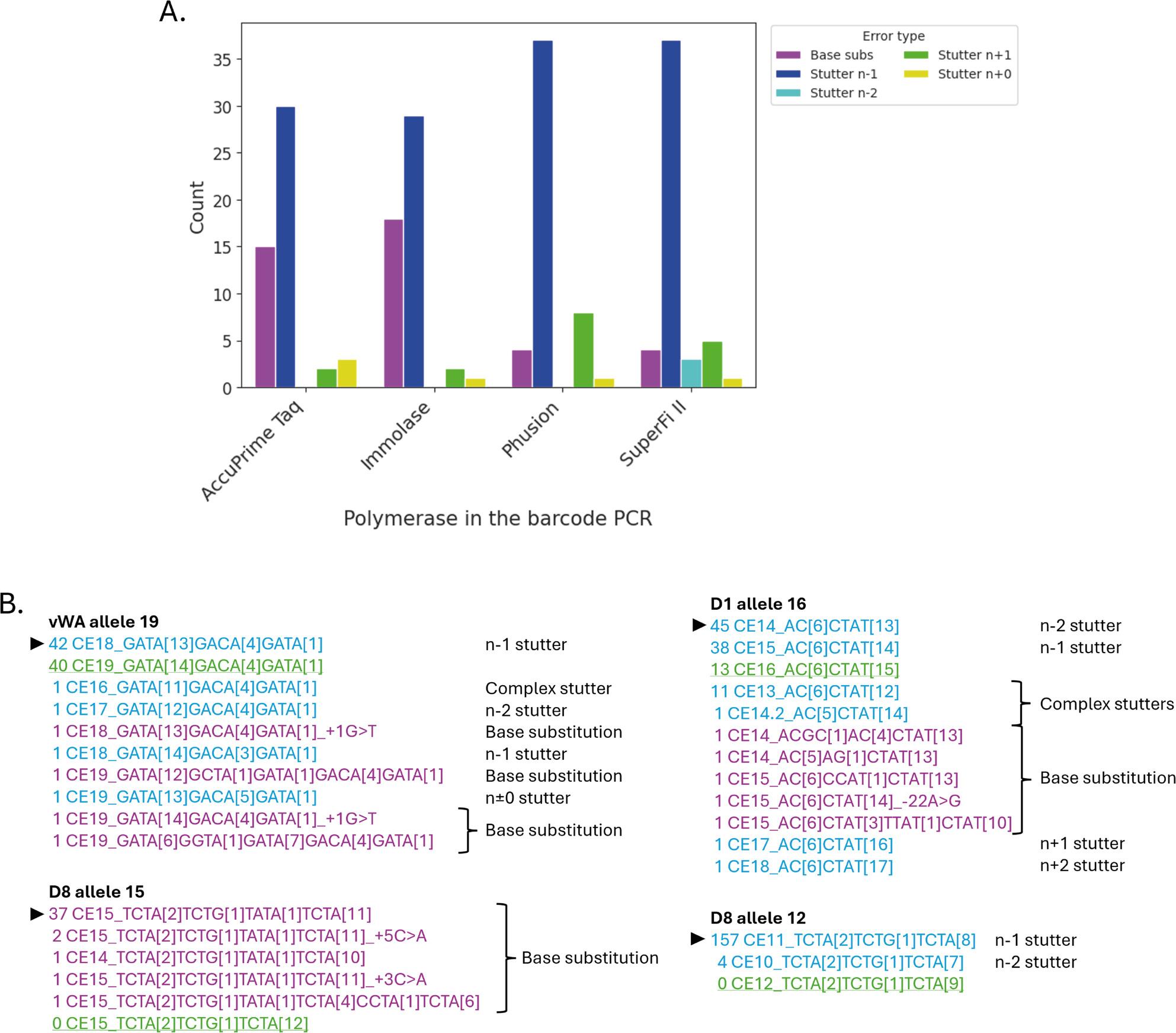
Feature values for UMI families with 40 or more members that provided correct consensus read for the different polymerases applied in the adaptor PCR. The boxplots show median values, the first and third quartiles and the whiskers 1.5 interquartile ranges, dots represent outliers. **A** Proportions of members within the UMI family, identical to the consensus sequence, i.e., purity. **B** Numbers of different sequence variants in the family, divided by the total number of members, i.e., proportion n variants. **C** Examples of UMI families. These families gave correct consensus reads, but with stutter ratios above 0.5, with the correct sequence underlined (green) and the proposed cause of error indicated in blue (stutter artefacts) and purple (base substitutions). The consensus read is indicated by an arrow. Sequences are given in STRnaming format. **D** Proportions of members one repeat unit shorter than the consensus read, i.e., proportion minus one. **E** Proportions of members two repeat units shorter than the consensus sequence, i.e., proportion minus two. **F** Proportions of members one repeat unit longer than the consensus sequence, i.e., proportion plus one. SuperFi II was used in the barcoding PCR when studying the effects of different DNA polymerases on adaptor PCR. For SuperFi II *n* = 40,732, Phusion HS II *n* = 51,782, AccuPrime Pfx *n* = 13,602, AccuPrime Taq HF *n* = 38,544, ExTaq HS *n* = 46,498 and Immolase *n* = 25,531

To study PCR errors emanating from individual amplicons, five features were used to describe UMI families with correct consensus reads for different DNA polymerases in adaptor PCR (Fig. 5). The mean purity, i.e., fraction of reads identical to the consensus sequence, was the highest for AccuPrime Taq HF and ExTaq HS at 82% and the lowest for AccuPrime Pfx at 69%. All DNA polymerases had UMI families that only contained the correct sequence (reaching a purity of 100%), except for AccuPrime Pfx with 98% purity as the highest value (Fig. 5A). The mean proportions of n variants (i.e., sequence variants) were considered equivalent for the polymerases with 0.16 for SuperFi II, Phusion, AccuPrime Pfx and Immolase and 0.14 for AccuPrime Taq HF and ExTaq HS. A value closer to 0 indicates fewer read variants within the UMI family, which is ideal. Values varied between 0.01 and 0.64 for individual UMI families across polymerases (Fig. 5B).

AccuPrime Pfx gave the highest mean proportions of stutter artefacts within the UMI families, with 11.5% n-1 stutter, 1.3% n-2 stutters, and 6.3% *n* + 1 stutters. These results were consistent with the overall proportions of incorrect STR reads and consensus reads generated by AccuPrime Pfx (Fig. 5D-F and D-F). SuperFi II (with proofreading activity and considered high fidelity) and Immolase (without proofreading activity) had similar mean proportions of the three stutter variants within the UMI families (8.8% versus 9.6% n-1 stutters; 0.7% versus 0.8% n-2 stutters; and 2.9% versus 3.0% *n* + 1 stutters, respectively). Phusion HS II, AccuPrime Taq HF and ExTaq HS gave the lowest mean proportions of stutters at 6.2%, 6.2% and 5.5% n-1 stutters, 0.4%, 0.4% and 0.3% n-2 stutters and 1.4%, 1.0% and 1.1% *n* + 1 stutters, respectively (Fig. 5D-F).

## Discussion

The evaluated DNA polymerases were chosen as representatives of enzymes with different activities and biochemical characteristics that possibly affect the degree of stutter artefacts and single-base substitutions. UMI families with incorrect consensus reads were studied to learn about errors formed in the initial PCR cycles, i.e., with genomic DNA as template. This revealed substantial differences in proportions of base substitutions between enzymes. Specifically, between the proofreading polymerases (SuperFi II and Phusion HS II, below 0.55% of other errors) and the polymerase preparations that either contained a separate 3’-5’ exonuclease (AccuPrime Taq HF, 1.7% other errors) or lacked proofreading activity (Immolase, 2.5% other errors). These findings coincide with the fidelities of the polymerases, which are 9X and likely around 1X compared to Taq polymerase for AccuPrime Taq HF and Immolase, respectively. Phusion HS II has 52X and SuperFi II 300X fidelity versus Taq polymerase. Thus, this outcome was expected due to the direct relationship between base substitutions and polymerase fidelity, where the misincorporation of bases is more common in amplicons made by polymerases lacking 3’-5’ exonuclease proofreading activity [39, 40].

In this study, we examined tetranucleotide STRs with different repeat motifs, but the findings should be relevant also for other repeat structures similar to the observations by Loh et al. [27]. There, optimization of the pre-sequencing workflow resulted in lowered stutter levels across the multiple motif lengths investigated. Here, stutters were seen both as incorrect consensus reads and incorrect members within UMI families with correct consensus reads. This implies that stutters were formed both in the initial cycles where genomic DNA serves as template, and in later cycles where STR amplicons are used to make more STR amplicons. Previous reports have suggested somewhat conflicting explanations for stutter formation, stating that the presence of a DNA-binding domain or high-fidelity properties may reduce the frequency of stutters [41] or that fidelity may not have a direct relationship with stutter risk, as slippage errors occur through other mechanisms than base substitutions [22, 42]. Platinum SuperFi DNA polymerase, an earlier version of SuperFi II, has previously been reported to result in lower stutter levels than five other commercial thermostable DNA polymerases, including both high fidelity enzymes and Taq-based ones, when analyzing STRs with capillary electrophoresis detection [22]. None of these polymerases were included in the present study. Here, SuperFi II yielded the highest proportions of stutters of the tested polymerases. The differences between the present study and Yamanoi et al. [22] may be partly due to the higher complexity and resolution in our sequencing method compared to single-plex analysis with capillary electrophoresis. Capillary electrophoresis only visualizes artefacts of different size. Also, the work made to improve Platinum SuperFi and develop SuperFi II, aimed at improving e.g., PCR inhibitor tolerance and long-range PCR compared to the previous version, may have led to unwanted side effects causing more stutters. Interestingly, when studying errors from individual template molecules, it was evident that both SuperFi II and Immolase, which are two enzymes with distinct characteristics, yielded very similar proportions of the systematic stutter artefacts. Hence, our results indicate that fidelity is not the main driver in stutter formation.

Different batches of all reagents and kits and different types of thermal cyclers were used at the two laboratories included in the study. In barcoding PCR, the ramp rate was 6.0 °C/s at laboratory A and 3.5 °C/s at laboratory B. With the exception of SuperFi II, no or minor differences in stutter frequencies between the laboratories were seen for the DNA polymerases. For SuperFi II, however, the stutter proportions differed somewhat. Since the only clear difference was seen for SuperFi II, this effect was likely due to differences between the applied SuperFi II batches and not to any general differences in e.g., library preparation or instrumentation. The different PCR ramp rates were not expected to affect stuttering, in part due to the long annealing time (6 min) that should minimize the impact of a quicker temperature change, and no such general effect was seen. By including two laboratories and two batches of each tested polymerase in the study, we have attempted to incorporate as much of the normal variation as possible. This, in turn, should provide more generally interpretable results than if one laboratory and one set of reagents were used.

The consensus reads generated reflect the STR amplicons produced during the early PCR cycles, i.e., in the barcoding PCR, where genomic DNA is the main template. The proportion of n-1 stutters varied between 0.48% and 4.4% for individual samples and different DNA polymerases. Phusion HS II showed the lowest variation between samples, from 1.0% to 2.0% n-1 stutter. This is consistent with previous reports suggesting that a DNA-binding domain may reduce stutter formation for mononucleotide repeats, likely through increased processivity [42]. However, the poorer performance of SuperFi II, which has the same general characteristics as Phusion HS II, indicates that other properties may be important for stuttering. The 7kD dsDNA-binding domain Sso7d from *Sulfolobus solfataricus*, which has no sequence preference, has previously been shown to improve the processivity of Pfu polymerase by 9-fold, from an average of 6 nucleotides to 55 nucleotides per binding event, through the fusion of Pfu polymerase and Sso7d [43]. Based on the available information from the manufacturer, it is likely that SuperFi II has a similar processivity as Phusion HS II, reported to be between 40 and 60 nucleotides per binding event. Still, SuperFi II gave substantially higher levels of stutters, indicating that improved processivity through the addition of a DNA-binding domain may not solely decrease the risk for stuttering. Likely, several different DNA polymerase characteristics affecting the stability of the ternary complex and extension kinetics impact the probability of stutter formation. Apart from DNA-binding domains, the bacteriophage T7 thioredoxin binding domain and the polypeptide thioredoxin have been shown to substantially reduce stutter artefacts through improved DNA binding via a conformational change of the polymerase [24].

When applying different polymerases in the barcoding PCR, substantial differences between enzymes were observed regarding numbers of raw reads as well as STR amplicon yields. Applying a polymerase that generates high STR amplicon yields, while also minimizing artifacts and errors, is especially important when applying UMI labelling using PCR, since the low concentrations of enzyme and primers in the barcoding PCR place increased demands on the DNA polymerase. Here, it was apparent that the barcoding PCR conditions were extremely challenging for some enzymes. This may be due to that manufacturers mainly optimize their DNA polymerase-buffer systems for high performance in regular PCR applications with excess of all reagents in the initial cycles.

It has previously been suggested that the usage of UMIs in library preparation reduces or even removes the need for high-fidelity polymerases [5]. There, it was argued that other characteristics such as amplicon yield are more important than fidelity, since the errors may be corrected informatically using the UMIs. UMIs are indeed effective for removal of erroneous amplicons, which is also seen in the present study. In sequencing applications that are only affected by single-base substitutions UMIs will likely provide high-quality data also with polymerases with low fidelity. However, the analysis of STR markers comes with the added challenge of systematic stutter artefacts. According to our findings, a combination of UMIs and an effort to minimize the generation of stutters biochemically provides a low overall impact of artifacts, thus enabling optimal limits of detection. When applied in the barcoding PCR, SuperFi II and Phusion HS II, both high-fidelity, proofreading polymerases with 3’ to 5’ exonuclease activity and DNA-binding domains, resulted in high STR amplicon yields. SuperFi II, however, gave substantially larger proportions of stutters than Phusion HS II, also after UMI-based consensus read generation. Ideally, a DNA polymerase to be used in STR analysis should provide both high STR amplicon yields and a low level of systematic stutter artefacts. A previous version of the polymerase has been shown to give both high amplicon yields and accurate sequencing results [8, 9], further strengthening the case for this family of enzymes in certain applications.

AccuPrime Taq HF, a blend of Taq DNA polymerase and a proofreading enzyme with accessory proteins for improved PCR fidelity, yield and selectivity showed inconsistent performance with a high STR amplicon yield of around 50% for six samples, but with below 20% for the other 18 samples. This polymerase was previously shown to provide improved amplicon yields for GC-rich regions, in combination with a lowered extension temperature [44]. AccuPrime Pfx, ExTaq HS and Immolase performed poorly in the barcoding PCR in terms of STR amplicon yield, and AccuPrime Pfx and ExTaq HS were thus excluded from evaluation regarding frequency of stutter artefacts and base substitution. AccuPrime Pfx has 26X fidelity compared to Taq polymerase due to its 3’-5’ exonuclease activity. Also, it has accessory proteins that improve the primer-template binding. ExTaq HS is a mix of Taq polymerase and a free exonuclease. The fidelity of ExTaq HS is 4.5X compared to Taq. Immolase does not have 3’-5’ exonuclease activity and its origin is a trade secret. It is chemically inactivated prior to starting the PCR. The other DNA polymerases in the study are inactive at room temperature through binding of antibodies or affibodies mediated. The heat activation prevents non-specific primer extension during PCR setup.

In the adaptor PCR, where STR amplicons serve as template, we observed high yields for all the evaluated polymerases. Compared to barcoding PCR, the different enzymes resulted in more uniform coverage and more similar error distributions. This is in part due to the UMIs that remove some (but not all) systematic and random errors. Other reasons for the smaller variation between polymerases in adaptor PCR is that STR amplicons are more accessible as templates compared to genomic DNA and that all reagents are in excess. Altogether, choosing an optimal DNA polymerase is less critical in the adaptor PCR than the barcoding PCR.

## Conclusions

We show that sequencing in combination with UMIs can be utilized to gain a deeper understanding of how DNA polymerase characteristics affect the generation of random and systematic polymerization errors in vitro. Two high-fidelity polymerases with DNA-binding domains, SuperFi II and Phusion HS II, both produced high STR amplicon yields and low levels of base substitution error rates through efficient amplicon generation from genomic DNA, compared to AccuPrime Taq HF and Immolase, which lack binding domains. The low concentrations of polymerase and primers in the barcoding PCR put high demands on enzyme performance. Apparently, the high fidelity or the DNA-binding domain of these polymerases make them suitable for this task. The SuperFi II and Phusion HS II polymerases have sub-units with 3’-5’ exonuclease activity. Seemingly, these are more efficient in reducing base substitutions than the free 3’-5’ exonuclease in AccuPrime Taq HF, likely due to the built-in proximity between the respective active sites. Immolase lacks 3’-5’ exonuclease activity altogether and is thus incapable of removing erroneously incorporated bases.

Overall, Phusion HS II gave the lowest proportions of stutters irrespective of whether genomic DNA or STR amplicons served as the initial template. Phusion HS II gave substantially less stutters of different types compared to SuperFi II, although they have similar general characteristics. Thus, high fidelity and increased processivity through a DNA-binding domain may not directly control the formation of stutters. This was further corroborated by the fact that polymerases lacking 3’-5’ exonuclease activity or DNA-binding domains gave similar or lower stutter levels than SuperFi II. Identifying a DNA polymerase that produces low levels of stutters and base substitutions may enable the detection of DNA from minor contributors in mixed forensic traces and low-level disease variants in clinical diagnostics. Combining such a polymerase with UMIs that remove erroneous amplicons bioinformatically will further improve the limit of detection. This study highlights the importance of an increased understanding of DNA polymerase function and how this can influence the quality of the sequencing results, especially when analyzing complex parts of the human genome such as STR markers.

## Supplementary Information

Supplementary Material 1.

## Data Availability

The sequencing data supporting the conclusions of this article are freely and openly available in the NIST Public Data Repository with the unique identifier: 10.18434/mds2-4088. Python scripts are available on request to Johannes Hedman at johannes.hedman@ple.lth.se.

## Declarations

### Disclaimer

Points of view in this document are those of the authors and do not necessarily represent the official position or policies of the U.S. Department of Commerce. Certain commercial software, equipment, instruments, and materials are identified in order to specify experimental procedures as completely as possible. In no case does such identification imply a recommendation or endorsement by NIST, nor does it imply that any of the materials, instruments, or equipment identified are necessarily the best available for the purpose.
