# Supplementary material for: DNA polymerase characteristics influence noise levels in sequencing of short tandem repeats
Source: BMC Genomics. 2026 May 26;27:507. doi: 10.1186/s12864-026-12985-4 (PMC13214446; doi:10.1186/s12864-026-12985-4)
Supplement: Supplementary file 1 — Supplementary Material 1. [file 12864_2026_12985_MOESM1_ESM.docx]

## Supplementary Figures

##
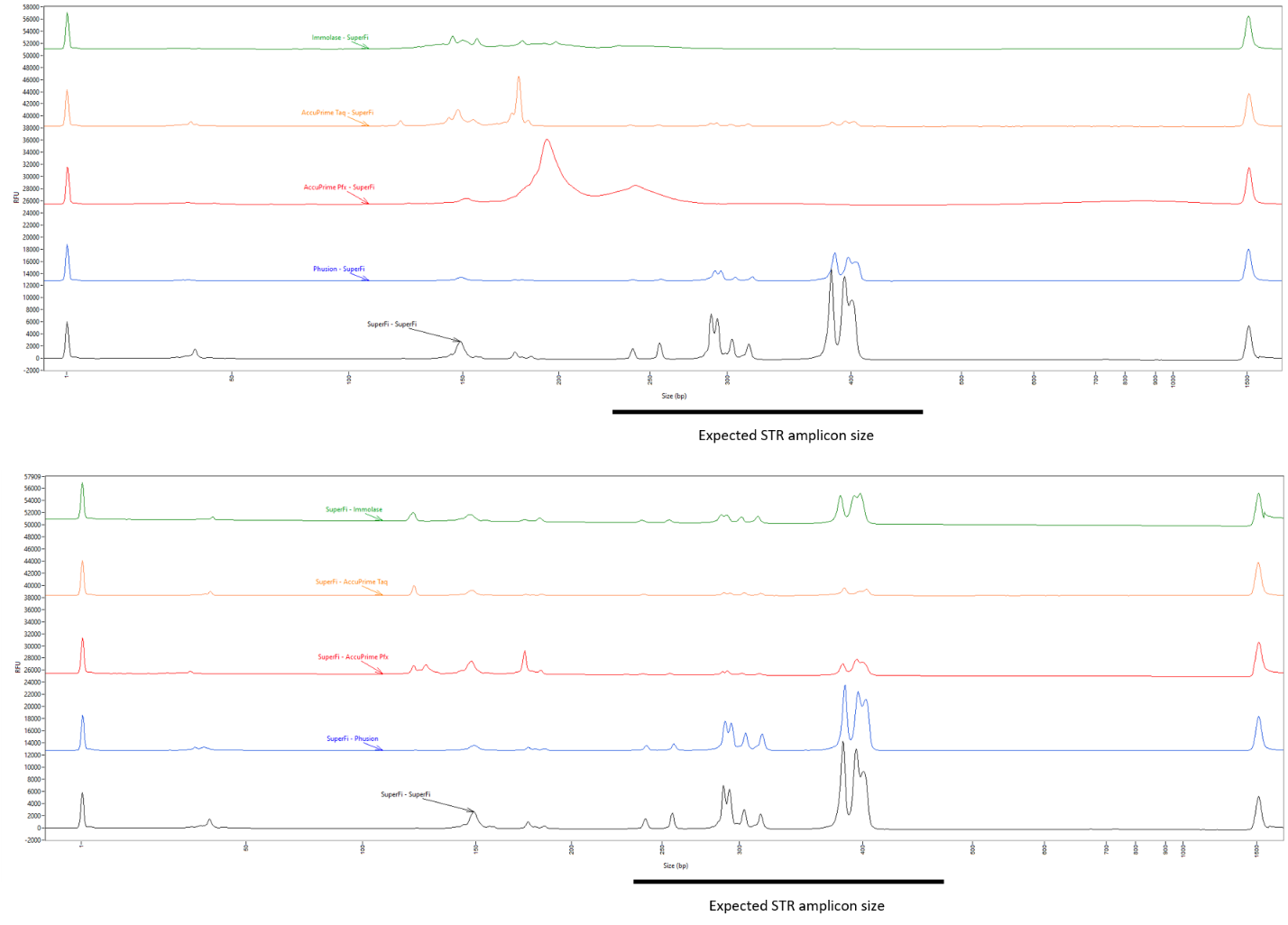


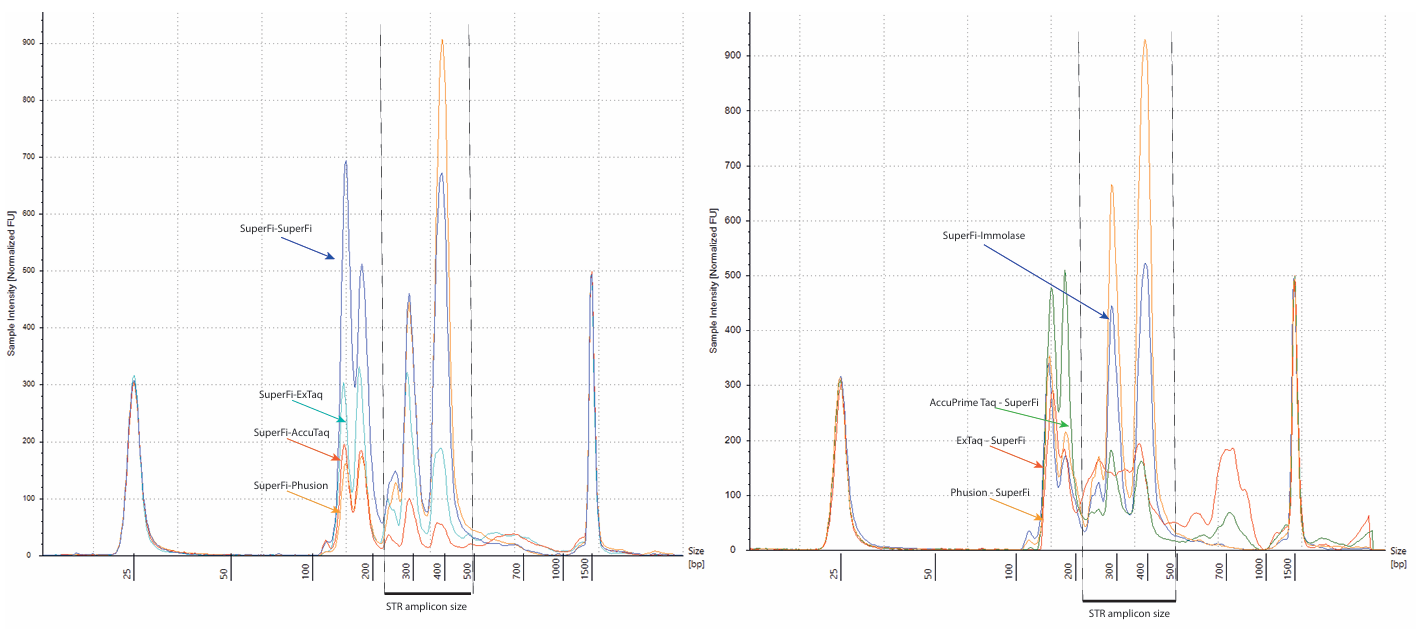


Figure S1: Examples of Fragment Analyzer System 5200 (Laboratory A) and TapeStation 4150 (Laboratory B) results for some samples before pooling of libraries. STR markers are expected between approximately 200 and 500 bp.

##
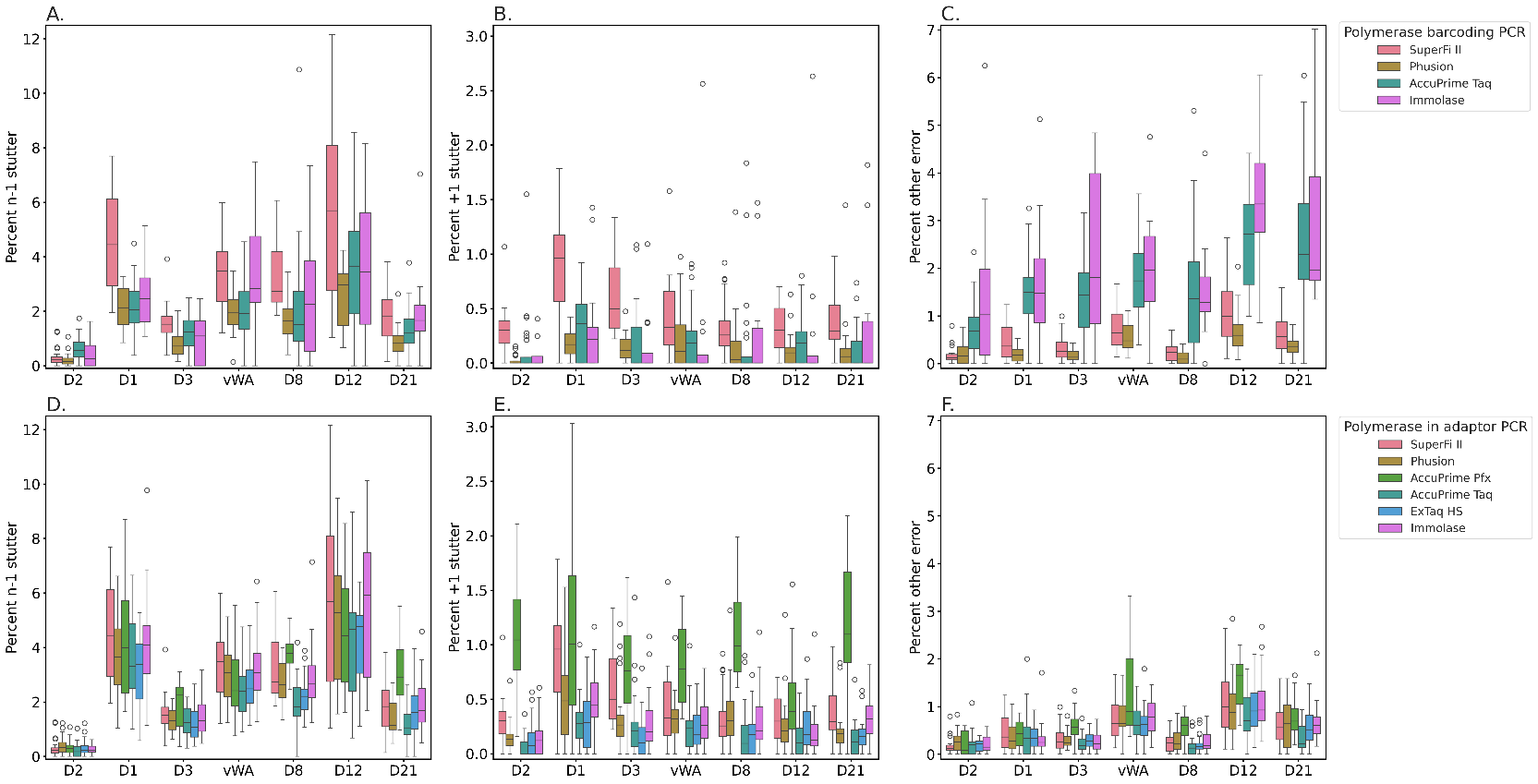


Figure S2: Proportion of artefacts and errors after consensus read generation, divided per STR marker for polymerases applied in the barcoding PCR (A, B and C) and in the adaptor PCR (D, E and F). A. and D. n-1 stutter ratio, B. and E. n+1 stutter ratio and C. and F. proportion of other errors. The boxplots show median values, the first and third quartiles and the whiskers 1.5 interquartile ranges, dots represent outliers.

Supplementary Tables

Table S1: Concentrations of samples determined with Qubit in ng/µL before pooling of the libraries, ranging from lowest concentrations to highest. Left column includes samples from Laboratory A and right column includes samples from Laboratory B.

| **Polymerase in barcoding PCR** | **Qubit concentration range (ng/µL)** | |
| --- | --- | --- |
| SuperFi II | 1.83, 2.39 | 3.83, 7.00 |
| Phusion HS II | 2.73, 3.27 | 2.91, 6.92 |
| AccuPrime *Pfx* | 5.52, 7.12 | - |
| AccuPrime *Taq* HF | 0.987, 1.78 | 2.34, 9.20 |
| Ex*Taq* HS | - | 2.70, 4.36 |
| Immolase | 3.56, 4.68 | - |
| **Polymerase in adaptor PCR** | **Qubit concentration (ng/µL)** | |
| Phusion HS II | 2.05, 3.28 | 3.33, 5.28 |
| AccuPrime *Pfx* | 1.04, 1.32 | - |
| AccuPrime *Taq* HF | 0.463, 1.03 | 1.36, 3.00 |
| Ex*Taq* HS | 1.41, 2.97 | 2.70, 4.36 |
| Immolase | 1.03, 1.72 | 2.20, 5.35 |
